# Temperature Field and Temperature Effects for Concrete Box Girder Bridges Based on Monitoring Data and Numerical Simulation

**DOI:** 10.3390/s25165036

**Published:** 2025-08-13

**Authors:** Mengxiang Zhai, Hongyin Yang, Bin Li, Jing Hao, Weihua Zhou, Hongyou Cao, Zhangjun Liu

**Affiliations:** 1 School of Civil Engineering and Architecture, Wuhan Institute of Technology, Wuhan 430073, China; 2 State Key Laboratory of Bridge Intelligent and Green Construction, Wuhan 430034, China; 3 Capital Construction Department, Wuhan Textile University, Wuhan 430073, China; 4 School of Civil Engineering and Architecture, Wuhan University of Technology, Wuhan 430070, China

**Keywords:** concrete box girder bridge, health monitoring, temperature field, temperature gradient, temperature effect, numerical simulation

## Abstract

The temperature field distribution and temperature effects of concrete box girder bridges were found to be critical to their long-term service safety. Based on long-term structural health monitoring data, the temperature field and temperature effects of a curved continuous concrete box girder bridge in Wuhan were investigated. A finite element model of the temperature field was established through the combined application of finite element software. Extreme weather files were constructed to analyze the bridge’s temperature field and temperature effects. To enhance data reliability, wavelet analysis was employed for denoising the monitoring data. The results indicate a strong correlation between girder temperature and ambient temperature. Under solar radiation, significant vertical temperature differences and certain lateral temperature differences are observed within the concrete box girder. The accuracy of the finite element model was validated through comparison with measured data. Temperature field models featuring the most unfavorable vertical and transverse temperature gradient distribution patterns for concrete box girder bridges under extreme weather conditions in the Wuhan region were established. A distinct temperature difference not covered by specifications exists at the webs and bottom slabs of the bridge. Strong correlations were observed between both pier–girder relative displacement and bottom slab stress with the girder temperature.

## 1. Introduction

With the progressive development of urbanization and growing traffic demands in China [1,2], elevated urban interchange bridges have become crucial for the improvement of urban traffic conditions [3]. Among these, concrete box girder bridges account for a large proportion [4,5,6], owing to their excellent structural performance and comparatively low construction cost [7,8,9].

Concrete box girder bridges are continuously subjected to complex natural environmental influences, including solar radiation and air temperature variations [10,11,12]. Due to the poor thermal conductivity of concrete material, nonlinear temperature distributions are formed within the concrete box girders under various thermal actions [13,14,15]. Consequently, different structural parts experience distinct thermal states, leading to thermal deformation [16]. When these deformations are subjected to internal and external constraints, significant thermal stresses are induced [17]. These thermal stresses may even exceed stresses caused by external live loads and are recognized as a primary cause of cracking in concrete bridges, potentially compromising the safety of concrete box girder bridges [18,19]. Current research is primarily focused on the empirical modeling of vertical temperature gradients [20,21,22], while the characteristics of lateral temperature differences and their influence on structural response have received considerably less attention [23,24,25]. Furthermore, although China encompasses diverse climate zones [26,27,28], the temperature gradient models used for bridge structures are still those uniformly specified in design codes, the applicability of which requires further investigation.

Early research on the temperature effects of concrete box girder bridges was primarily focused on the characteristics of temperature field distribution and structural deformation patterns induced by such effects. Roberis-Wollman et al. [29] developed empirical formulas for predicting the most unfavorable temperature field in concrete box girder bridges and calculated the relationship between the temperature gradient and temperature deformation. Li, D.N. et al. [30] observed significant seasonal displacements in concrete box girder bridges under temperature influence and concluded that the provisions for temperature gradient patterns in the Canadian Design Code are overly conservative. Lee, J.H. et al. [31] analyzed the lateral and vertical temperature gradients in box girder bridges under different environmental conditions based on long-term monitoring data. Additionally, the deflection of the structure induced by lateral and vertical temperature differences was calculated using one-dimensional beam theory.

With advancements in monitoring technology and numerical simulation methods, analyses of structural response characteristics to thermal effects were initiated from multidimensional perspectives. Taysi, N. et al. [32] investigated the temperature distribution and its variation in concrete bridges under the action of temperature through field tests and 3D finite element software. Moravcik, M. et al. [33] provided a brief overview of the temperature load on bridge structures and conducted a comparative analysis between the measured vertical temperature gradients in concrete box girder bridges and those specified in the Eurocode. Mathew, C.S. et al. [34] established a finite element model of a concrete box girder bridge and applied the temperature load to calculate the temperature effects on the bridge under the action of temperature. Systematic advances were achieved in recent research across multiple dimensions, including field validation, model development, and code evaluation. These advances significantly enhanced the theoretical depth and engineering applicability of thermal effect studies.

The remainder of this paper is organized as follows: Section 2 presents an overview of the arrangement of monitoring points on the box girder bridge, and temperature field monitoring data was analyzed. Section 3 details the methodology for establishing the finite element model of the temperature field, and the model accuracy was validated. Temperature gradients in both vertical and transverse directions of the bridge were predicted under constructed extreme climatic conditions and were compared with those specified in design codes. Section 4 presents the processing of temperature effect monitoring data, and a detailed analysis was conducted on the relationship between temperature effects and temperature. Section 5 summarizes the main concluding remarks.

## 2. Measurement Point Layout and Monitoring Data Analysis

### 2.1. Measurement Point Layout

**Figure 1:**
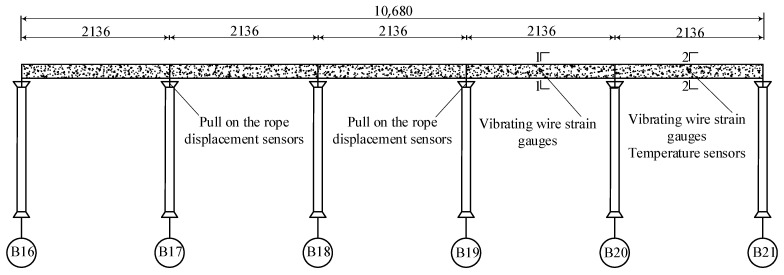
Concrete box girder bridge span arrangement (unit: cm).

This study focuses on a five span (5 × 21.36 m) curved continuous concrete box girder bridge in Wuhan City. The span arrangement of the bridge is illustrated in Figure 1. The bridge is oriented north–south with a curve radius of 650 m. Double-column piers are adopted at both ends, while a single-column pier is utilized at the midspan. The main girder is a constant section, single-cell concrete box girder with a height of 1.5 m. The top slab is 10 m wide, and the bottom slab is 5 m wide. Both cantilever slabs extend 2.5 m outward, with their thickness varying linearly from 0.15 m to 0.4 m, respectively. The main girder is constructed with C40 concrete. The top slab is overlaid with a C40 concrete leveling course, and the deck surface is paved with asphalt concrete.

**Figure 2:**
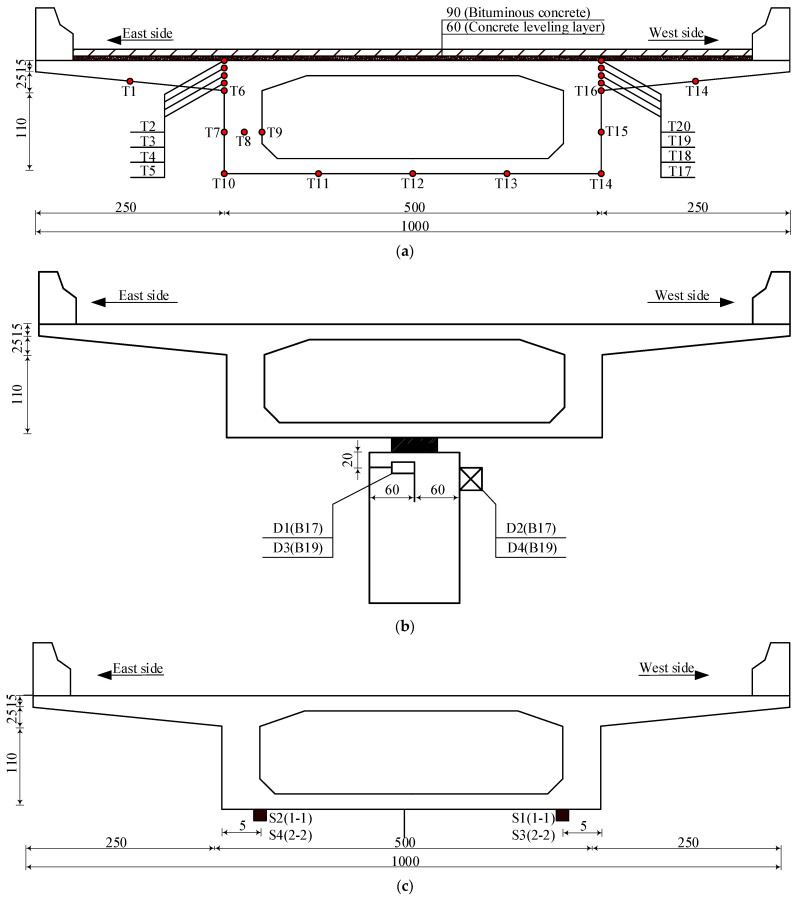
Layout of monitoring points on concrete box girder: (**a**) layout of temperature monitoring points (unit: cm); (**b**) layout of pier-girder relative displacement monitoring points (unit: cm); and (**c**) layout of stress monitoring points at girder bottom (unit: cm).

Based on the temperature field distribution characteristics of the concrete box girder bridge and the requirements for temperature effect analysis, the measuring points of this bridge were arranged as illustrated in Figure 2: Temperature sensors of type M222-PT1000 (T-1 to T-21) were deployed on the fifth span, establishing 21 temperature measurement points across the concrete box girder cross-section. Pull rope displacement sensors of type LVDT-RS-50 (D-1 to D-4) were installed atop piers B17 and B19, comprising four measurement points, where D-1 and D-3 monitored longitudinal displacement, while D-2 and D-4 monitored transverse displacement to track pier-to-girder relative displacements. Vibrating wire strain gauges of type BGK-4080 (S-1 to S-4) were positioned at cross-section 1-1 of the fourth span and cross-section 2-2 of the fifth span, forming four measurement points for monitoring bottom flange stresses at both sections. The data acquisition was performed at 10 min intervals.

### 2.2. Analysis of Temperature Field Monitoring Data

**Figure 3:**
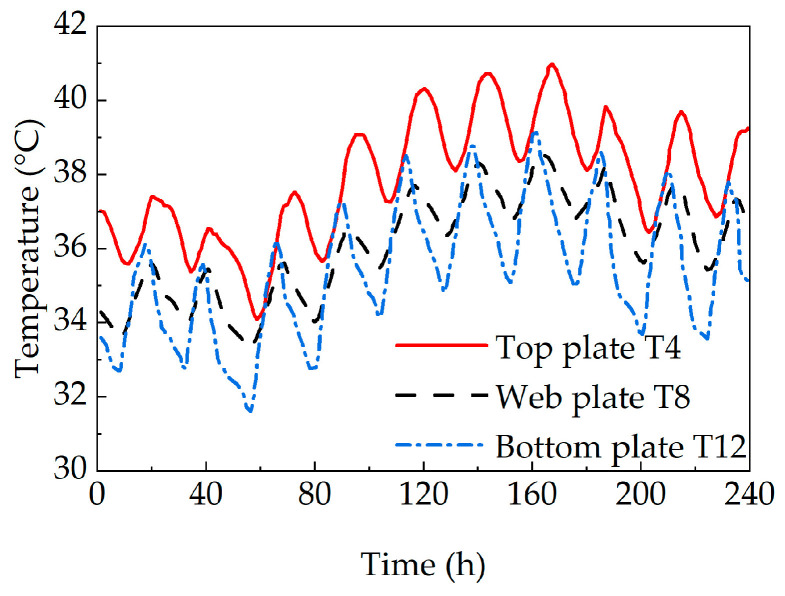
Temperature variation curves at central monitoring points of structural parts in the concrete box girder bridge.

Temperature monitoring data from 22 July to 31 July 2019 was selected to analyze the temperature variations in the concrete box girder bridge. The results are presented in Figure 3. As observed from the figure, within the same section of the concrete box girder bridge, the top slab, web, and bottom slab exhibit similar temperature variation patterns. The temperature of the top slab is significantly higher than that of the web and bottom slab. During the heating phase, the bottom slab temperature exceeds the web temperature, while during the cooling phase, the web temperature is higher than the bottom slab temperature. Moreover, a significant vertical temperature difference exists in the section of the concrete box girder bridge, with a daily maximum temperature difference of 5.6 °C.

**Figure 4:**
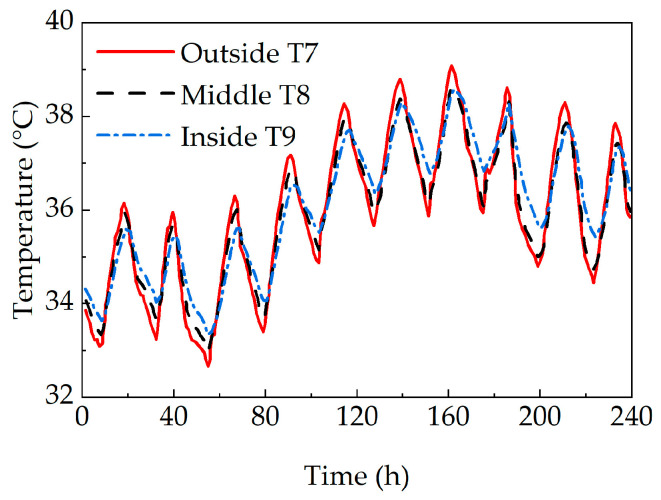
Temperature time-history variations through the thickness of the web.

Figure 4 displays the temperature variation curves of the monitoring points on the east web of the concrete box girder bridge. As observed, the temperature variation range at the outer surface monitoring point is significantly larger than that at the inner surface monitoring point. Moreover, a lateral temperature difference exists across the web, with a daily maximum temperature difference of 1.3 °C.

During the heating stage, the concrete on the outer surface of the web is directly exposed to solar radiation. Due to the poor thermal conductivity of concrete, heat is transferred slowly to the inner concrete, resulting in a delayed temperature rise internally. Furthermore, as the inner surface of the web is located in a relatively enclosed environment, a lateral temperature difference is observed. During the cooling stage, heat is simultaneously dissipated by the outer web concrete to the surrounding atmosphere and transferred inward to the inner concrete. Consequently, the temperature of the outer concrete is reduced more rapidly than that of the inner concrete.

## 3. Temperature Field of Concrete Box Girder Bridge

### 3.1. Temperature Field Model of Concrete Box Girder Bridge

#### 3.1.1. Establishment of Temperature Field Model

A simulation methodology employing combined finite element software was adopted to analyze the temperature field of the concrete box-girder bridge. Thermal analysis software TAITHERM is recognized for its facilitated thermal modeling approach and efficient thermal solving capabilities. However, TAITHERM is limited to constructing simple built-in models and cannot be used for complex bridge geometric modeling. In contrast, finite element software ANSYS 2020 R1 enables convenient geometric modeling of bridge structures. File format incompatibility between TAITHERM and ANSYS models necessitated the implementation of HYPERMESH 2020.2 software as a format converter, achieving seamless data interoperability between the platforms.

Initially, the geometric model of the box girder bridge was established in ANSYS software based on its structural information. The geometric model was subsequently imported into HYPERMESH software for meshing and format conversion, where it was exported into a format compatible with TAITHERM 2020.2 software. Finally, the meshed geometric model was imported into TAITHERM software, bonded into an integrated unit, and subjected to parameter definition. Weather data files were then imported, and the temperature field of the concrete box girder bridge was calculated.

**Figure 5:**
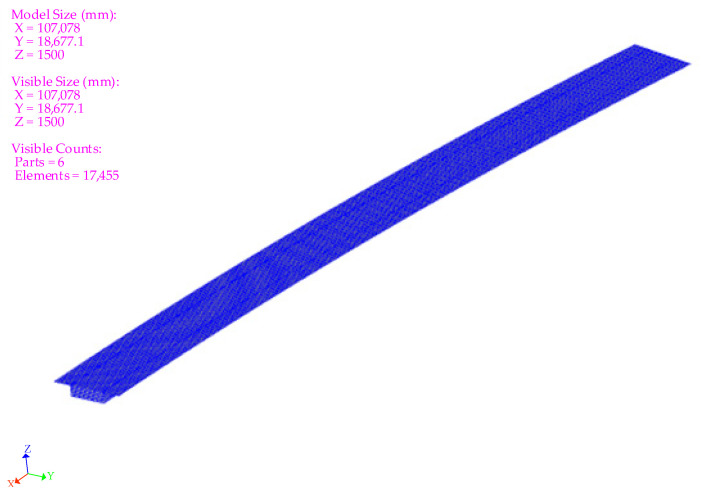
Temperature field model of concrete box girder bridge.

The temperature field model for the concrete box girder bridge is depicted in Figure 5. The model was discretized into 17,455 elements, with the top slab, bottom slab, web, and diaphragms partitioned according to their actual thicknesses. All components were assigned to concrete material properties. An asphalt pavement layer with an actual thickness of 9 cm was added to the top slab. Through analysis of the simulated values and the monitored values, the calibrated material parameters were determined, as summarized in Table 1.

Based on the actual conditions of the bridge, the geographic information and surface boundary conditions of the box girder bridge were configured. Subsequently, weather files containing meteorological parameters such as solar radiation, ambient temperature, wind speed, humidity, and cloud cover were imported. This enabled the calculation of the temperature field distribution in the concrete box girder bridge.

#### 3.1.2. Verification of Temperature Field Model

**Figure 6:**
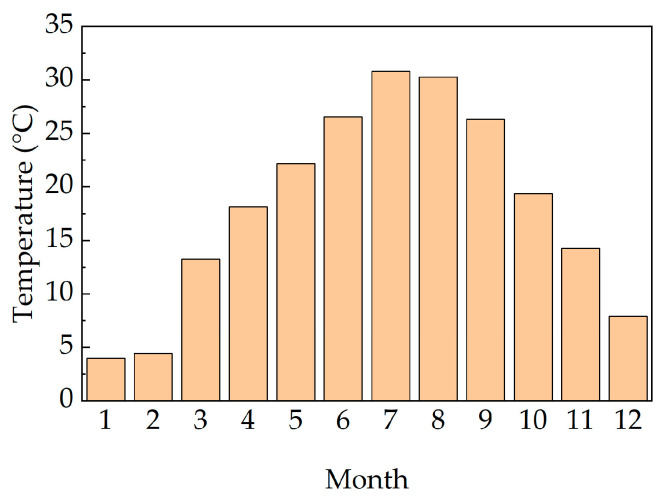
2019 monthly average temperatures.

This study utilized monitoring data for the entire year of 2019 to calculate the temperature field of the concrete box girder bridge on a monthly basis. Figure 6 presents the monthly average temperatures in Wuhan City during 2019. As observed, January exhibited the lowest monthly average temperature, while July recorded the highest monthly average temperature. Therefore, monitoring data from January and July was selected to validate the temperature field model of the concrete box girder bridge.

**Figure 7:**
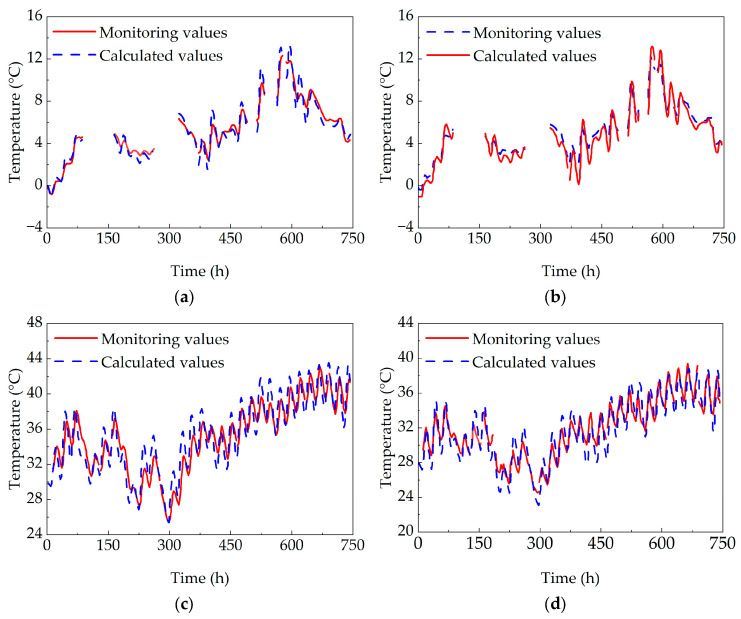
Comparison between temperature monitoring value and calculated value of concrete box girder bridge: (**a**) comparison of top slab temperature in January; (**b**) comparison of bottom slab temperature in January; (**c**) comparison of top slab temperature in July; and (**d**) comparison of bottom slab temperature in July.

A comparative analysis was conducted between the model-calculated temperature values and the field-monitored temperature values of the box girder bridge. A comparison between monitored and calculated values for selected measurement points (top slab T4 and bottom slab T12) is presented in Figure 7.

During the monitoring period, partial temperature monitoring data for January was lost, attributed to potential influences from equipment malfunctions or external interference on the monitoring system. Nevertheless, the accuracy of the temperature field model was still able to be validated. As shown in the figure, the calculated temperature values and monitored temperature values at the monitoring points, both the top slab and bottom slab of the concrete box girder bridge, are comparable in magnitude and exhibit highly consistent variation trends. All absolute errors are within 3 °C, demonstrating that the temperature field model of the concrete box girder bridge, established based on weather files and finite element software, is reasonable and accurate.

### 3.2. Temperature Gradient Patterns of Box Girder Under Extreme Weather Conditions

#### 3.2.1. Determining Extreme Weather Conditions

According to the specification [35], the design reference period for highway bridges and culverts is 100 years, so the return period of meteorological parameters is also considered as 100 years. Significant temperature gradients are generally observed in concrete box girder bridges during hot summer conditions. If constructing extreme weather files for Wuhan City, the randomness of meteorological parameters needs to be considered. The meteorological parameters that affect the temperature field of concrete box girder bridges under a sunny environment are mainly solar radiation, ambient temperature, and wind speed.

According to the method for determining outdoor air calculation parameters in specification [36], the hourly representative values of the most unfavorable solar radiation in Wuhan City can be determined. Unlike solar radiation and ambient temperature, wind speed changes have no obvious pattern, and the distribution of wind speed values has large dispersion. The daily average wind speeds from July to September 2019 were adopted as the hourly representative values for the most unfavorable wind speed.

The random and independent nature of the temperatures satisfied the distribution conditions of extreme value statistical theory. The daily maximum temperatures recorded in Wuhan over the past decade were employed as samples. Modeling and calculation were performed using the Type I extreme value distribution; this method was widely utilized for predicting temperature extremes. Its exponential decay characteristics exhibited a strong alignment with the statistical patterns observed in the daily maximum temperatures. As shown in Table 2, the most severe temperature for Wuhan is calculated based on extreme value statistical theory.

The Type I extreme value distribution function of the extreme value statistical theory is as follows:

(1) $$F(T)=P(T\leq t)=e^{-\beta }$$

(2) $$\beta =e^{\frac{-(T-\alpha )}{\lambda }}$$

where F(T) is the probability that the temperature is less than or equal to t, λ is the scale parameter, and α is the position parameter.

Define the variables for Equation (1):

(3) $$\mu =\frac{T-\alpha }{\lambda }$$

The relationship between return period G and distribution function F(T) is:

(4) $$G=\frac{1}{1-F(T)}$$

(5) $$F(T)=1-\frac{1}{G}$$

Therefore, the relationship between μ and the return period G is:

(6) $$\mu =-\ln[-\ln(1-\frac{1}{G})]$$

From Equation (3), the linear function of T and μ is:

(7) T=λμ+α

The values of α and λ are derived, respectively, as follows:

(8) $$\lambda =\frac{\sqrt{6}}{\pi }S,\;\;\alpha =M+\lambda \gamma$$

where γ is the Euler–Mascheroni constant, γ = 0.5772, M is the sample mean, and S is variance.

The sample mean is 38.28, and the sample variance is 1.337. Combining the above equations, the calculation yields that the daily maximum temperature for a return period of 100 years is 44.5 °C. Meanwhile, based on the daily maximum temperatures within the design reference period, the analysis obtains the hourly representative values of the most unfavorable ambient temperature.

Using the hourly representative values of the most unfavorable solar radiation, ambient temperature, and wind speed among other meteorological parameters, the extreme weather files for Wuhan City within the design reference period were constructed. Combined with the temperature field model of the concrete box girder bridge, the most unfavorable temperature gradients of the concrete box girder bridge under extreme weather conditions occurring for 5 consecutive days were calculated.

#### 3.2.2. Prediction of Vertical Temperature Gradient

When the temperature difference between the maximum temperature on the upper surface of the bridge structure and the minimum temperature in the vertical direction of the girder reaches the maximum value, the vertical temperature distribution can be regarded as the most unfavorable vertical temperature gradient.

**Figure 8:**
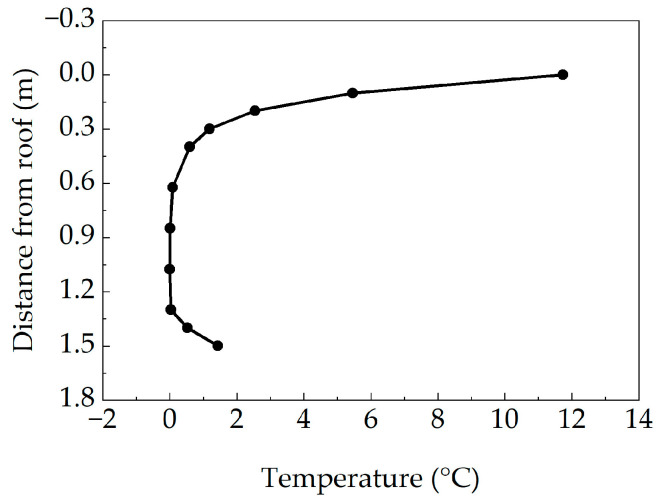
Vertical temperature difference distribution curve of box girder section.

Under a sunny environment, vertical temperature differences are generated along the girder height direction in the concrete box girder section, and certain lateral temperature differences also exist in the web thickness direction. The constructed extreme weather files were imported into the TAITHERM software to calculate the temperature field of the concrete box girder bridge, obtaining the most unfavorable temperature gradients of the box girder bridge under extreme weather conditions. Figure 8 shows the vertical temperature difference distribution curves at different depths along the vertical direction of the box girder section.

As seen from the figure, in the concrete box girder section along the vertical direction, there are significant temperature difference variations within certain depth ranges from the top slab and bottom slab, with the maximum temperature difference being 11.73 °C. Meanwhile, the temperature difference values at the middle web are relatively small. The temperature difference distribution in the upper part of the concrete box girder bridge resembles an exponential function form. The temperature difference distribution in the lower part of the concrete box girder bridge exhibits linear variation.

The vertical temperature difference distribution of the box girder bridge was fitted in the form of a piecewise function, predicting the most unfavorable vertical temperature gradient distribution function for concrete box girder bridges in Wuhan City within the design reference period as follows:

(9) $$\left\{\begin{array}{l} T_{y}=11.7e^{-7.8y},\;0\leq y\leq H-0.2\\ T_{y}=7.1y-9.3,\;H-0.2\leq y\leq H \end{array}\right.$$

where $T_{y}$ is the temperature difference at the vertical calculation point of the box girder section, y is the distance from the calculation point to the top surface of the box girder top slab, and H is the height of the box girder section.

The coefficient of determination (R^2^) serves as a key metric for evaluating the goodness of fit in regression models. The coefficient of determination was employed to evaluate fitting performance in this study, resulting in a computed value of 0.9967. Close agreement between the fitted function and observed data is indicated by this result.

**Figure 9:**
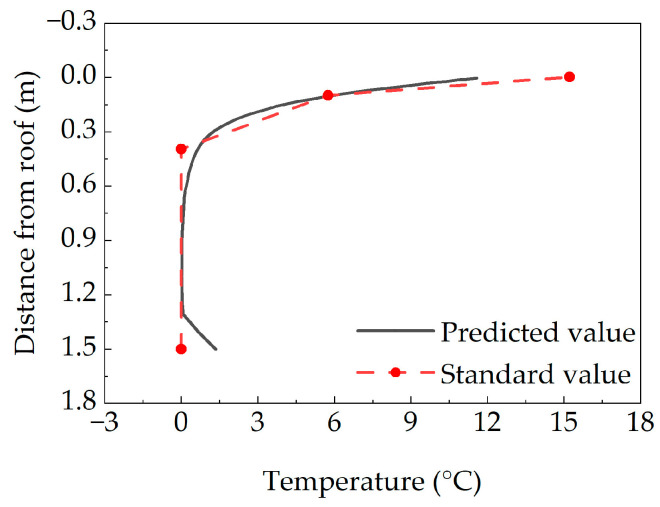
Comparison of vertical temperature gradient.

The value of the vertical temperature gradient for bridge structures in China is primarily calculated using a double broken line distribution pattern [35]. The predicted most unfavorable vertical temperature gradient for the concrete box girder bridge is compared with the vertical temperature gradient specified in the current Highway Bridge Code, with the comparison results presented in Figure 9.

As observed from the figure, the temperature gradient distribution in the upper part of the concrete box girder bridge exhibits an exponential function form, differing from the double broken line distribution in the Highway Bridge Code. At the top slab of the concrete box girder, the predicted vertical temperature gradient values were all found to be within the code-specified limits. However, at the lower sections of the webs and at the bottom slab, predicted temperature gradient values were observed to exceed the code-specified limits. At the bottom slab, the predicted temperature gradient was 1 °C higher than the code-specified limit, while at the top slab, it was 3 °C lower than the code-specified limit. Significant temperature gradients were identified at the upper web sections and the bottom slab of the box girder bridge. As no relevant provisions are included in the current highway bridge design codes, attention is required from the relevant personnel. Therefore, practical studies should specifically analyze the vertical temperature gradient of bridge structures by considering the actual bridge conditions and weather conditions.

#### 3.2.3. Prediction of Lateral Temperature Gradient

**Figure 10:**
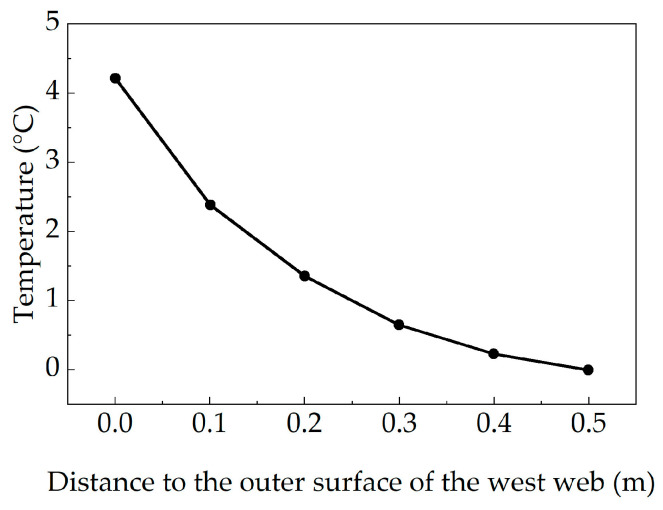
Lateral temperature difference distribution curve of box girder web.

Under a sunny environment, due to the shading effect of the flange plate on the web, the lateral temperature difference distribution in the web of this box girder bridge is less pronounced than the vertical temperature difference distribution. The west web of the concrete box girder bridge is more significantly affected by the ambient temperature. Therefore, the lateral temperature difference in the west web was analyzed, using the temperature at the innermost point of the same section at half girder height of the west web as the reference point. Figure 10 shows the lateral temperature difference distribution curves for the west web of the box girder bridge.

As observed from the figure, the lateral temperature difference distribution in the west web of the concrete box girder bridge also resembles an exponential function form, the maximum temperature difference is 4.21 °C. The lateral temperature difference data of the box girder web were fitted to predict the most unfavorable lateral temperature gradient distribution function for concrete box girder bridges in Wuhan City within the design reference period as follows:

(10) $$T_{x}=4.2e^{-6.3x}$$

where $T_{x}$ is the temperature difference at the lateral calculation point of the web in the box girder section, and x is the distance from the calculation point to the outer surface of the box girder web.

The goodness of fit was evaluated by the coefficient of determination (R^2^), which was calculated as 0.9927, indicating an excellent agreement between the fitted function and the observed data.

## 4. Analysis of Temperature Effect of Concrete Box Girder Bridge

### 4.1. Processing of Temperature Effect Monitoring Data

During the structural health monitoring process of bridge structures, anomalies or missing data in the monitoring data may be caused by sensor noise, environmental factors, and human factors. Wavelet analysis possesses the capability for localized time-frequency analysis of signals. It was found to be particularly advantageous for denoising data containing multiple frequency components [37], exhibiting temporal variations, and being non-stationary. The Symlets wavelet basis function is well suited for preserving critical signal features while effectively removing noise. Consequently, the Symlets wavelet basis function was employed for denoising the original monitoring data, with a decomposition level of 7.

**Figure 11:**
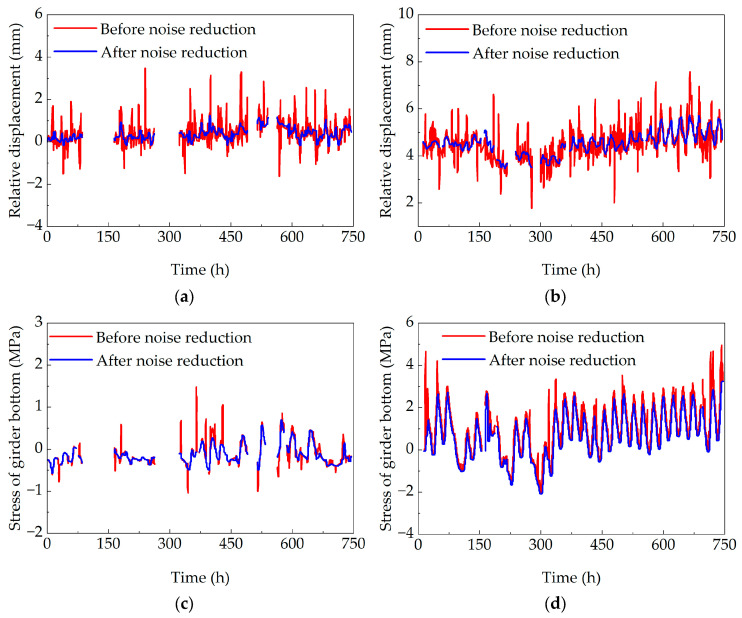
Noise reduction diagram of original monitoring data: (**a**) noise reduction diagram of pier and girder relative displacement in January; (**b**) noise reduction diagram of pier and girder relative displacement in July; (**c**) stress noise reduction diagram of girder bottom in January; and (**d**) stress noise reduction diagram of girder bottom in July.

This study selected the displacement monitoring point at pier B17 and the stress monitoring point at the bottom west side of section 1-1 of the concrete box girder bridge for January and July 2019. The original monitoring data from these points were subjected to denoising, with the processed results presented in Figure 11. The denoised data exhibits relatively smooth variations and significantly reduced spikes, demonstrating that the wavelet analysis method can effectively eliminate noise interference in original monitoring data.

### 4.2. Relationship Between Temperature Effects and Temperature

**Figure 12:**
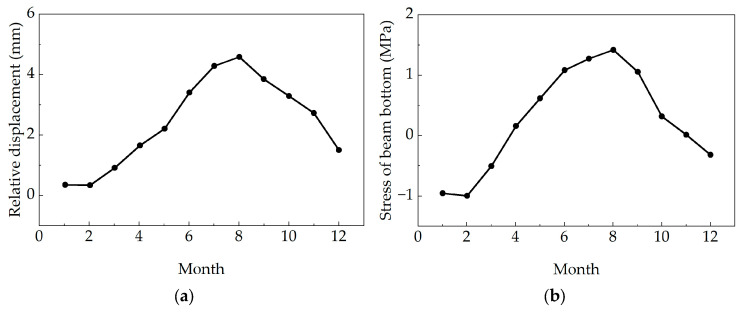
Monthly average values of pier girder relative displacement and girder bottom stress: (**a**) monthly average value of relative displacement; and (**b**) monthly average girder bottom stress.

The monitoring data of relative pier girder displacement and bottom slab stress in bridge structures can reflect the operational state of the bridge. This study selected the 2019 monitoring data to investigate the variations in relative pier girder displacement and bottom slab stress, as shown in Figure 12.

As observed, the temporal trends of both relative pier girder displacement and bottom slab stress exhibit distinct seasonal variation characteristics. The measured values for both parameters are larger in summer and smaller in winter, showing similarity to the variation trend of the girder temperature.

**Figure 13:**
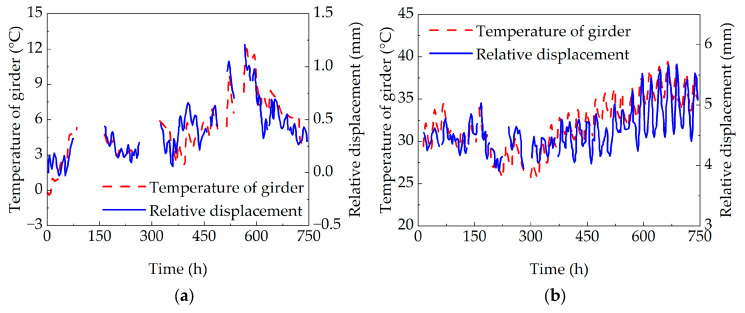
Comparison between relative displacement of pier and girder and temperature of beam body: (**a**) comparison of relative displacement and temperature of pier and girder in January; and (**b**) comparison of relative displacement and temperature of pier and girder in July.

To thoroughly investigate the correlation between the pier girder relative displacement and girder temperature in a concrete box girder bridge, displacement monitoring data from pier B17 of the bridge for January and July 2019 were selected. After noise reduction processing of the original monitoring data, the hourly variations in the pier girder relative displacement and girder temperature are presented in Figure 13.

As shown in the figure, the pier girder relative displacement varies with changes in girder temperature and exhibits a certain time lag compared to the variation in girder temperature. Correlation analysis was performed on the monitoring data using SPSS 27 statistical data analysis software, revealing a positive correlation between pier girder relative displacement and girder temperature variation in the concrete box girder bridge. The Pearson correlation coefficient was 0.79 in January and 0.81 in July, indicating a strong correlation between pier girder relative displacement and girder temperature.

**Figure 14:**
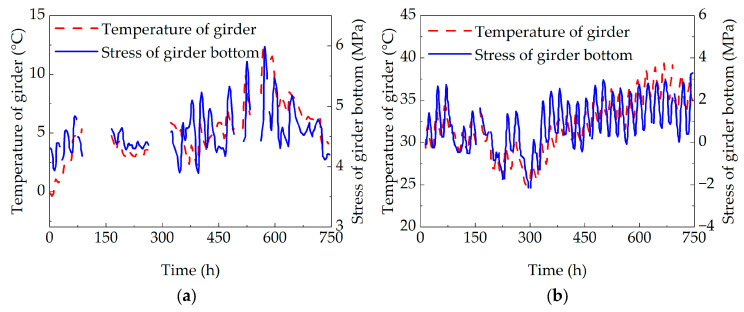
Comparison of girder bottom stress and girder temperature: (**a**) comparison of girder bottom stress and temperature in January; and (**b**) comparison of girder bottom stress and temperature in July.

Stress monitoring data from the west side of the bottom surface at section 1-1 of the concrete box girder bridge for January and July 2019 were selected. After noise reduction processing of the original monitoring data, the variations in girder bottom stress and girder temperature over time are presented in Figure 14.

As shown in the figure, the girder bottom stress in the concrete box girder bridge follows a similar trend to the girder temperature variation and also exhibits a certain time lag. Correlation analysis of the monitoring data was conducted using SPSS statistical data analysis software, revealing a positive correlation between girder bottom stress and girder temperature variation. The Pearson correlation coefficient was 0.74 in January and 0.77 in July, indicating a strong correlation between girder bottom stress and girder temperature.

## 5. Conclusions

This study focused on a curved concrete continuous box girder bridge in Wuhan, incorporating real-time monitoring data of girder temperature, pier girder relative displacement, and bottom slab stress. A finite element model for simulating the bridge temperature field was established, with an analysis of temperature gradient distribution under extreme weather conditions. The main conclusions are as follows:

1. Based on temperature monitoring data of the concrete box girder bridge, the distribution characteristics of the bridge temperature field were investigated. Annually, the top slab experiences the largest temperature variation, followed by the web, with the bottom slab exhibiting the smallest variation. Significant vertical temperature differences were identified within the same cross-section, along with certain lateral temperature gradients.
2. The simulated temperature field showed good agreement with the monitoring data, validating the accuracy of the established model. This demonstrates that the combined finite element simulation approach can reliably capture the thermal behavior of concrete box girder bridges, can be extended to similar structural analyses.
3. Extreme weather conditions in Wuhan with a 100-year return period were constructed and used in conjunction with the temperature field model to predict the most unfavorable lateral and vertical temperature gradient distributions. The maximum vertical temperature difference is 11.73 °C, and the maximum lateral temperature difference is 4.21 °C. Comparisons with current highway bridge design specifications revealed that the specified values for the top slab are reasonable. However, unregulated yet significant temperature gradients were found in the upper web and bottom slab, indicating the need for potential specification refinement.
4. Wavelet analysis was employed to denoise the original monitoring data of pier girder relative displacement and bottom slab stress for the concrete box girder bridge, effectively reducing interference. The processed data demonstrated that the temporal variations in displacement and stress closely followed those of girder temperature, exhibiting strong correlations.

The impact of thermal loads on bridge structures is governed by multiple influencing factors, with significant variations exhibited across different bridge configurations. The temperature gradient distribution pattern predicted in this study applies solely to concrete box girder bridges in Wuhan. For concrete box girder bridges in other regions, local meteorological data must be collected and combined with corresponding bridge models to determine their temperature gradient distribution patterns.

## Figures and Tables

**Table 1:** Material parameter table.

Material	Density (kg·m^−3^)	Thermal Conductivity (W·m^−1^·°C^−1^)	Heat Capacity (J·kg^−1^·°C ^−1^)	Absorbency	Reflectivity
Concrete	2500	1.74	970	0.65	0.85
Asphalt	2300	0.7	860	0.88	0.93

**Table 2:** Daily maximum temperature for Wuhan in the recent 10 years.

Year	Maximum Temperature (°C)	Year	Maximum Temperature (°C)
2010	39.6	2015	36.4
2011	37.3	2016	38.4
2012	37.5	2017	39.7
2013	39.5	2018	38.6
2014	37.1	2019	38.7

## Data Availability

The data presented in this study is available upon request from the corresponding author.
